# Supplementary material for: Ethnicity, Social, and Clinical Risk Factors to Tooth Loss among Older Adults in the U.S., NHANES 2011–2018
Source: Int J Environ Res Public Health. 2022 Feb 18;19(4):2382. doi: 10.3390/ijerph19042382 (PMC8875070; doi:10.3390/ijerph19042382)
Supplement: Supplementary file 1 [file ijerph-19-02382-s001.zip › ijerph-1565287-supplementary.pdf]

Supplementary Table S1. Results of multivariate analysis on ethnicity group and number of missing tooth by age group.

**Age group**	**Ethnicity**	**Number of missing tooth**		
		**β**	**S.E.**	**p-value**
20~44	Hispanic	-1.25	0.16	<.001
	Non-Hispanic Black	-1.38	0.14	<.001
	Non-Hispanic Asian	-0.76	0.20	<.001
	Other Race - Including Multi-Racial	-0.67	0.25	0.007
	Non-Hispanic White	Ref.		
45~64	Hispanic	-1.62	0.34	<.001
	Non-Hispanic Black	0.58	0.27	0.033
	Non-Hispanic Asian	-0.71	0.43	0.101
	Other Race - Including Multi-Racial	1.01	0.59	0.087
	Non-Hispanic White	Ref.		
65~	Hispanic	-2.42	0.54	<.001
	Non-Hispanic Black	1.22	0.41	0.003
	Non-Hispanic Asian	-1.80	0.73	0.014
	Other Race - Including Multi-Racial	4.19	0.97	<.001
	Non-Hispanic White	Ref.	.	

Supplementary Table S2. Results of multivariate analysis on ethnicity group and number of missing tooth by income.

**Income (month)**	**Ethnicity**	**Number of missing tooth**		
		**β**	**S.E.**	**p-value**
\$0~2099	Hispanic	-2.49	0.37	<.001
	Non-Hispanic Black	-0.87	0.29	0.003
	Non-Hispanic Asian	-1.95	0.56	0.001
	Other Race - Including Multi-Racial	0.42	0.60	0.491
	Non-Hispanic White	Ref.		
\$2100~\$5399	Hispanic	-1.50	0.31	<.001
	Non-Hispanic Black	0.64	0.26	0.013
	Non-Hispanic Asian	-1.22	0.41	0.003
	Other Race - Including Multi-Racial	1.06	0.51	0.038
	Non-Hispanic White	Ref.		
\$5400~\$5399	Hispanic	-0.60	0.40	0.132
	Non-Hispanic Black	1.15	0.38	0.002
	Non-Hispanic Asian	-0.16	0.51	0.752
	Other Race - Including Multi-Racial	1.38	0.77	0.076
	Non-Hispanic White	Ref.		
\$8400~	Hispanic	0.30	0.37	0.424
	Non-Hispanic Black	0.60	0.33	0.068
	Non-Hispanic Asian	0.48	0.37	0.196
	Other Race - Including Multi-Racial	0.73	0.62	0.242
	Non-Hispanic White	Ref.		
